# Local Simvastatin for Hard-Tissue Healing After Third Molar Extraction: A Systematic Review

**DOI:** 10.1097/SCS.0000000000013261

**Published:** 2026-08-25

**Authors:** Vincenzo Ronsivalle, Paola Lo Giudice, Marco Cicciù, Fabio Mongelli

**Affiliations:** Department of General Surgery and Surgical-Medical Specialties, School of Dentistry, University of Catania, Catania, Italy

**Keywords:** Bone regeneration, simvastatin, socket healing, third molar, tooth extraction

## Abstract

**Background:**

After third molar extraction, the socket undergoes a physiological healing and remodeling process that influences postextraction bone fill. Because third molar surgery provides a relatively standardized clinical model for evaluating socket healing, several local adjuncts have been investigated to enhance osteogenesis and improve hard-tissue repair. Among these, simvastatin has attracted interest because of its reported osteopromotive, angiogenic, and antiresorptive effects. However, the available clinical evidence remains heterogeneous. The aim of this systematic review was to evaluate the effects of locally delivered simvastatin on hard-tissue healing and postoperative clinical outcomes in third molar extraction sockets.

**Methods:**

PubMed, Scopus, Web of Science, and LILACS were searched from inception to October 10, 2025. Clinical studies assessing local/topical simvastatin in mandibular or maxillary third molar sockets were included. Primary outcomes were postextraction hard-tissue healing and bone regeneration assessed by radiographic, tomographic, histologic, or histomorphometric parameters. Secondary outcomes included pain, swelling, trismus, soft-tissue healing, and postoperative complications. Because of marked heterogeneity, findings were synthesized narrative.

**Results:**

Ten studies were included. Most reported improved radiographic or tomographic indicators of hard-tissue healing in simvastatin-treated sockets, including higher gray-scale values, greater bone density, earlier mineralization, and improved trabecular microarchitecture. Clinical outcomes were less consistent, with variable effects on pain, swelling, and soft-tissue healing. Comparative studies showed mixed results.

**Conclusions:**

Local simvastatin may be associated with improved imaging-based indicators of hard-tissue healing after third molar extraction, but the evidence remains heterogeneous, methodologically limited, and less consistent for patient-centered outcomes.

Healing of the extraction socket after third molar removal remains a relevant clinical issue, particularly when the objective is to promote early bone regeneration and improve socket maturation. Following tooth extraction, the alveolar bone undergoes a physiological but clinically relevant remodeling process, with substantial horizontal and vertical dimensional changes occurring, especially during the early phases of healing.^1^,^2^ In this context, interest has grown in locally delivered adjuncts capable of enhancing osteogenesis and improving postextraction bone healing.

Third molar surgery represents a useful and relatively standardized clinical model for investigating socket healing and postextraction bone regeneration.^3^,^4^,^5^ Among the adjunctive materials proposed for this purpose, simvastatin has attracted increasing attention because of its reported capacity to promote mineralized tissue formation, vascularization, and socket healing when applied directly to extraction sites.^6^,^7^,^8^,^9^ Early evidence from extraction socket grafting suggested that simvastatin, when combined with a grafting material may improve early histologic healing by increasing newly formed mineralized areas and vascularity while reducing inflammatory infiltrate.^10^,^11^,^12^

From a biological perspective, postextraction healing involves clot stabilization, provisional matrix formation, woven bone deposition, and progressive remodeling of the socket walls, all of which may influence the quality and rate of bone fill within the extraction site.^13^,^14^ In this regard, third molar surgery represents a useful and relatively standardized clinical model for investigating postextraction socket healing and bone regeneration. Initial split-mouth studies evaluating different local formulations showed that both simvastatin powder and simvastatin gel were associated with progressive radiographic bone fill, although without significant differences between the 2 preparations.^2^ Subsequent randomized clinical trials comparing simvastatin with saline or conventional socket management generally reported higher radiographic gray-scale values and greater bone density in simvastatin-treated sites over follow-up periods ranging from weeks to months, supporting a positive effect on osseous regeneration after mandibular third molar removal.^3^,^15^,^16^,^17^,^18^ Additional tomographic and histologic investigations further suggested improvements in trabecular microarchitecture and socket quality, although these findings were not always statistically significant and, in some cases, improved hard-tissue healing appeared to be accompanied by greater early postoperative pain, swelling, or delayed soft-tissue recovery.^19^,^20^

The interest in simvastatin is biologically plausible, as statins have been shown to stimulate bone formation through increased BMP-2 expression and to promote bone repair at healing sites by enhancing osteoblastic activity while suppressing osteoclastic resorption.^21^,^22^ Comparative studies have also produced heterogeneous findings. Simvastatin showed more favorable early bone regeneration than melatonin in one split-mouth trial,^23^ and simvastatin-containing synthetic scaffolds performed better than similar scaffolds without the drug, although they remained inferior to deproteinized bovine bone mineral with collagen.^12^ Conversely, xenograft-based sticky bone demonstrated superior postoperative and radiographic outcomes compared with simvastatin in impacted mandibular third molar sockets.^12^,^24^ Taken together, the available literature suggests that topical simvastatin may be associated with improved postextraction hard-tissue healing after third molar extraction. However, the evidence remains difficult to interpret because of marked heterogeneity in carriers, formulations, comparators, follow-up durations, and outcome measures, as well as inconsistent findings regarding pain, swelling, and soft-tissue healing. Therefore, a systematic review focused specifically on the local use of simvastatin in third molar extraction sockets is warranted to critically synthesize the available evidence and clarify its potential value in promoting postextraction bone healing.

## METHODS

### Eligibility Criteria

The eligibility of the studies was assessed according to the Population, Intervention, Comparison, and Outcomes (PICO) framework.^25^

**(P)** The population consisted of human subjects undergoing mandibular or maxillary third molar extraction requiring postextraction socket healing assessment.

**(I)** The intervention consisted of the local/topical application of simvastatin into the extraction socket, either alone or combined with carriers, scaffolds, membranes, collagen sponges, gelfoam, bone substitutes, or other delivery systems.

**(C)** The comparison included spontaneous healing, placebo, standard socket management, carrier alone, other biomaterials, alternative regenerative materials, alternative pharmacologic adjuncts, or different local formulations of simvastatin.

**(O)** The primary outcomes were postextraction hard-tissue healing and bone regeneration, assessed through radiographic, tomographic, histologic, or histomorphometric parameters, including bone density, gray-scale values, bone fill, and trabecular features. Secondary outcomes included soft-tissue healing, postoperative pain, swelling, trismus, infection, alveolar osteitis, and other postoperative complications.

Only original clinical studies reporting conclusive postintervention data were considered eligible for inclusion. Randomized clinical trials, split-mouth studies, prospective comparative studies, and controlled pilot clinical studies were included. The exclusion criteria were as follows: (1) animal or in vitro studies; (2) case reports, case series, narrative reviews, systematic reviews, and meta-analyses; (3) conference abstracts, posters, editorials, and letters to the editor; (4) studies not published in English; (5) studies not involving tooth extraction sockets; (6) studies in which simvastatin was not locally delivered into the socket or extraction site; and (7) studies with insufficient or nonextractable outcome data.

### Search Strategy

We systematically searched Web of Science, PubMed, Scopus, and Lilacs for articles published from the inception until October 10, 2025. We followed the strategy reported in Supplemental Table 1, Supplemental Digital Content 1, https://links.lww.com/SCS/J976. The search strategy was developed by combining terms related to simvastatin with terms related to tooth extraction, extraction socket healing, socket preservation, and alveolar bone regeneration. In addition, a manual search of the reference lists of the included studies and relevant reviews was performed to identify any further eligible articles. This systematic review was conducted in accordance with the Preferred Reporting Items for Systematic Reviews and Meta-Analyses (PRISMA 2020) statement and followed the recommendations of the Cochrane Handbook for Systematic Reviews of Interventions.^26^,^27^ The review protocol was registered in PROSPERO CRD420261375414.

### Data Extraction

The data were extracted from each included study: (1) first author and year of publication; (2) country of origin; (3) study design; (4) number of patients and/or surgical sites; (5) characteristics of the extracted teeth; (6) type, concentration, dose, and carrier of simvastatin; (7) comparator intervention; (8) follow-up period; (9) methods used for outcome assessment; and (10) main clinical, radiographic, tomographic, and histologic findings.

### Data Synthesis

Because of the expected clinical and methodological heterogeneity among the included studies, a quantitative meta-analysis was not planned. The included studies differed substantially in study design, type of third molar treated, simvastatin formulation and carrier, comparator intervention, follow-up duration, and outcome assessment methods. Therefore, the findings were synthesized descriptively through a qualitative narrative approach.

The results were grouped according to the main domains of interest: (1) hard-tissue healing and bone regeneration outcomes, including radiographic, tomographic, histologic, and histomorphometry findings; and (2) clinical outcomes, including pain, swelling, trismus, soft-tissue healing, and postoperative complications. When appropriate, the findings were also interpreted according to the type of comparator used (spontaneous healing/carrier alone versus active regenerative interventions) and the follow-up period.

Given the variability in outcome measures and reporting methods across studies, no pooled effect estimates were calculated. The synthesis was therefore aimed at identifying consistent trends, differences across interventions and comparators, and the main sources of heterogeneity relevant to the clinical interpretation of the evidence.

### Quality Assessment

The methodological quality and risk of bias of the included studies were assessed independently by 2 reviewers. For randomized clinical trials, the revised Cochrane risk-of-bias tool for randomized trials (RoB 2) was used. For nonrandomized comparative studies, the risk of bias in nonrandomized studies of interventions (ROBINS-I) tool was applied. Disagreements were resolved by discussion, and when consensus was not achieved, a third reviewer made the final decision.

## RESULTS

### Study Selection

A total of 1938 records were identified through database searching. As illustrated by the PRISMA 2020 flow diagram, 159 duplicate records, 78 review articles, and 86 records removed for other reasons were excluded before screening. The remaining 1615 records underwent title and abstract screening, of which 1331 were excluded. Two hundred eighty-four reports were sought for retrieval, 23 were not retrieved, and 261 full-text articles were assessed for eligibility. Among these, 251 reports were excluded, mainly because they did not respond to the PICO question (n=185) or were off-topic (n=66). Finally, 10 studies were included in the present systematic review. The study selection process is summarized in the PRISMA 2020 flow diagram (Fig. 1).

**FIGURE 1:**
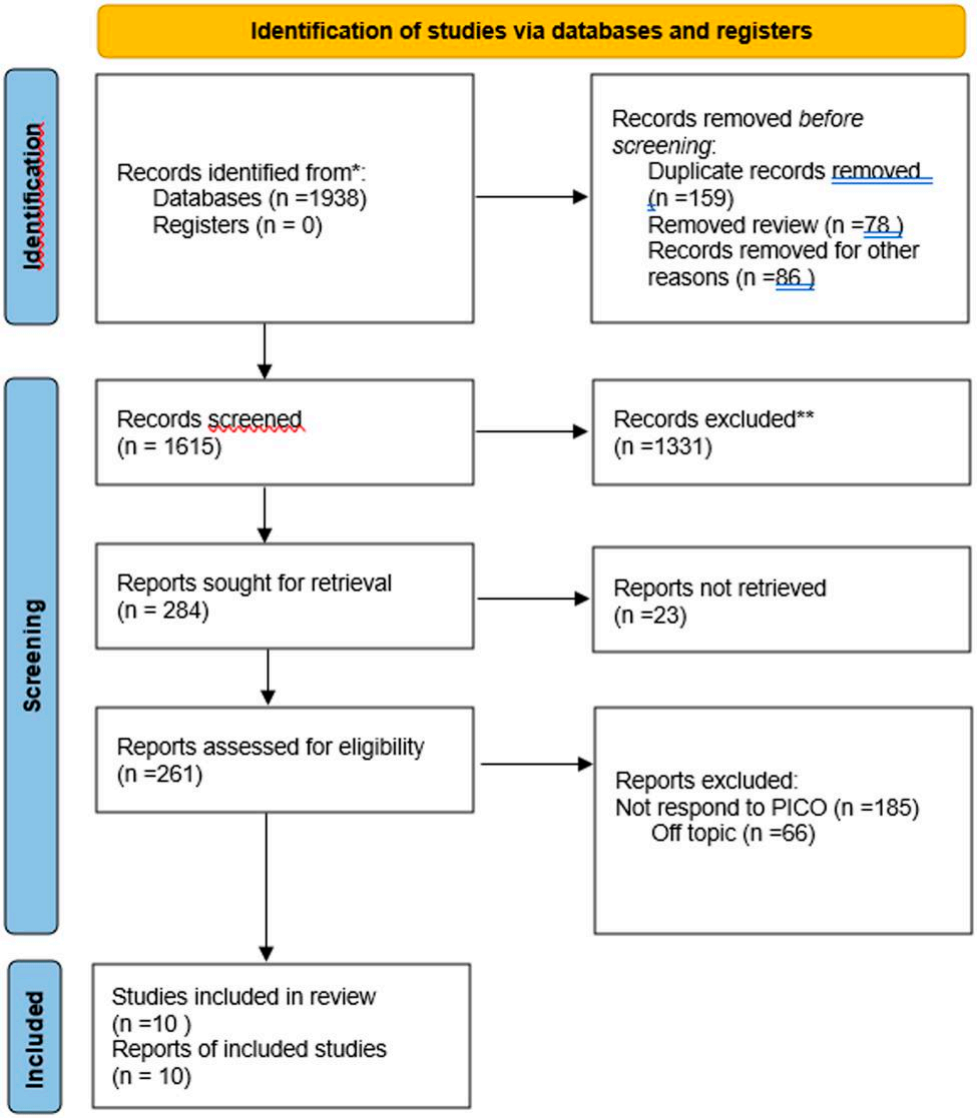
PRISMA flowchart.

### Study Characteristics

The included studies were published between 2015 and 2025 and were mainly conducted in India, with additional studies from Brazil and Iraq. Most investigations were prospective randomized split-mouth clinical trials, whereas one study was designed as a clinical pilot study and another as a prospective comparative randomized clinical study. The sample size ranged from 10 to 50 participants, although some authors also reported the number of extraction sockets or cases. Nearly all studies evaluated mandibular third molars, while one pilot randomized controlled trial assessed maxillary third molar sockets. In all included studies, simvastatin was locally applied into the extraction socket, most commonly at a dose of 10 mg, using carriers such as gelfoam, gelatin sponge, collagen sponge, or scaffold-based biomaterials. The comparator was generally spontaneous healing or the same carrier without simvastatin, although some studies compared simvastatin with melatonin, simvastatin gel, simvastatin powder, xenograft/sticky bone, or alternative scaffold materials. The main outcomes assessed across the studies were radiographic bone regeneration or bone density, usually by intraoral radiographs, gray-scale histogram analysis, or CBCT, together with clinical outcomes such as pain, swelling, wound healing, trismus, and postoperative complications. The follow-up period ranged from 3 months to 6 months in most studies. The main characteristics of the included studies are summarized in Supplemental Table 2, Supplemental Digital Content 2, https://links.lww.com/SCS/J977.

### Main Findings

#### Hard-Tissue Outcomes

Most included studies reported more favorable hard-tissue healing in simvastatin-treated sockets than in untreated or carrier-only control sites. The reported benefits mainly included higher radiographic gray-scale values, greater bone density, earlier mineralization, and improved socket microarchitecture.^15^,^16^,^17^,^18^,^19^ These findings were observed across different local delivery systems, including gelfoam, gelatin sponge, and collagen-based carriers.^15^,^16^,^17^,^18^,^19^ In the study directly comparing simvastatin powder and simvastatin gel, both formulations were associated with progressive bone fill over time, without significant intergroup differences.^2^

#### Clinical Outcomes

Clinical postoperative outcomes were less consistent across the included studies. Several studies reported no significant differences between simvastatin-treated and control sites in terms of pain, swelling, or soft-tissue healing.^2^,^15^,^16^,^17^ In contrast, some investigations described lower pain scores and improved early postoperative comfort in the simvastatin groups.^18^,^23^ However, one tomographic trial reported greater early pain, swelling, and delayed short-term mucosal healing despite improved bone microarchitecture at 3 months.^19^

### Comparative Findings

When compared with active alternative interventions, simvastatin showed mixed results. Simvastatin demonstrated more favorable early bone regeneration than melatonin in one split-mouth study.^23^ It also appeared to improve the performance of synthetic scaffolds compared with the same scaffold without the drug, although it did not outperform deproteinized bovine bone mineral with collagen in maxillary third molar ridge preservation.^12^ In another study, sticky bone was associated with superior postoperative and radiographic outcomes compared with simvastatin in impacted mandibular third molar sockets.^24^

### Quality Assessment and Risk of Bias

The domain-level risk-of-bias judgments are summarized in Figures 2 and 3. Nine randomized studies were assessed using the Cochrane Risk of Bias 2 (RoB 2) tool, whereas one nonrandomized comparative study was assessed using ROBINS-I, as prespecified in the Methods section.

**FIGURE 2:**
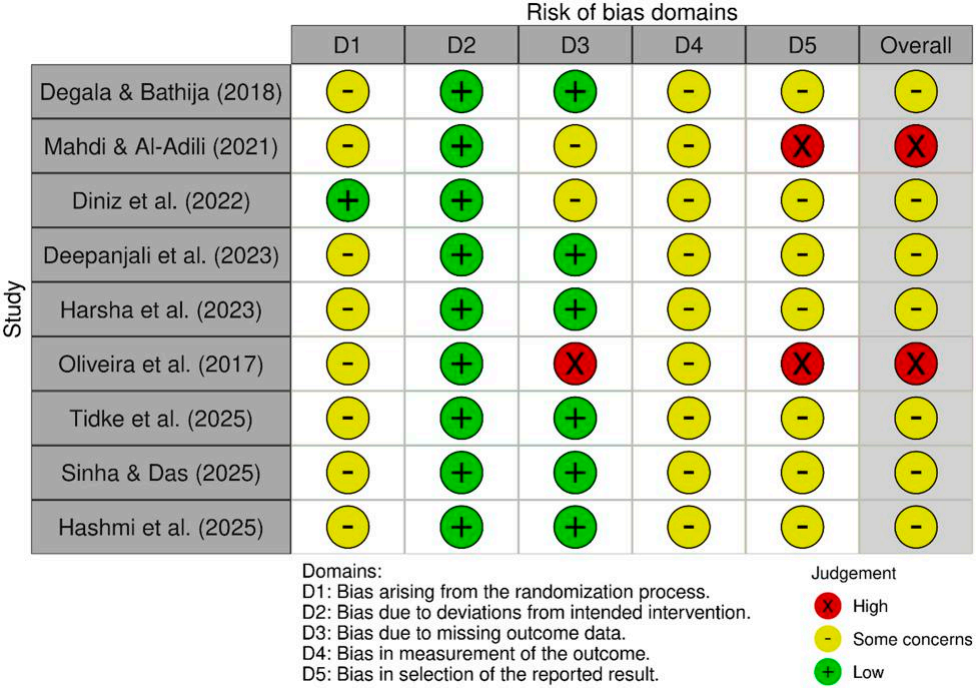
Assessment of risk of bias: RoB 2 tool.

**FIGURE 3:**
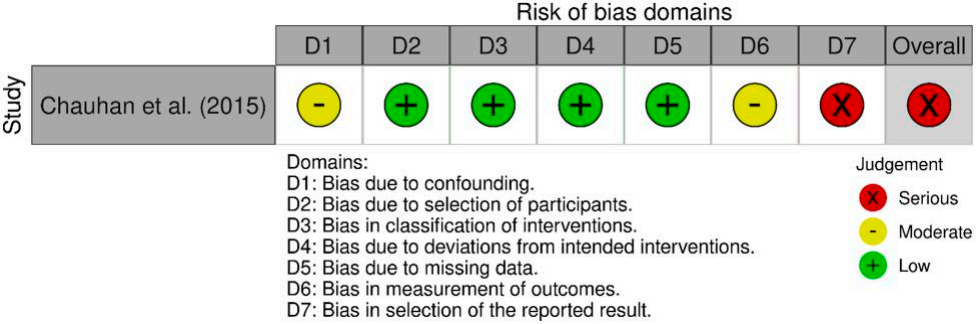
Assessment of risk of bias: ROBINS-I.

Among the randomized studies, most were judged as presenting some concerns overall, whereas 2 were considered at high risk of bias. The most frequent limitations involved the randomization process, measurement of the outcome, and selection of the reported result, mainly because of incomplete reporting of allocation procedures, limited blinding of outcome assessment, and insufficient detail on outcome reporting. Bias due to deviations from intended interventions was generally judged as low.

The nonrandomized comparative study showed a serious overall risk of bias, mainly because of concerns related to confounding, measurement of outcomes, and selection of the reported result. Bias due to confounding represented the main limitation in this study, whereas other domains were less consistently problematic.

Overall, the methodological quality of the included evidence was not negligible, and the findings should therefore be interpreted with caution.

## DISCUSSION

The present systematic review suggests that locally delivered simvastatin may be associated with improved postextraction hard-tissue healing after third molar surgery, particularly in terms of radiographic and tomographic indicators. Across the included studies, simvastatin-treated sockets frequently showed higher gray-scale values, greater bone density, earlier mineralization, and improved socket microarchitecture when compared with spontaneous healing or passive carriers alone.^15^,^16^,^17^,^18^,^19^,^23^ However, these findings should be interpreted cautiously, as they were derived mainly from surrogate imaging-based outcomes, whereas the evidence for clinically meaningful advantages remains less consistent. In this context, any apparent benefit of simvastatin should not be interpreted as evidence of a clear or uniform improvement in overall postoperative recovery.

The biological plausibility of these findings is supported by experimental evidence indicating that statins may upregulate BMP-2-related pathways and promote bone repair through a combined anabolic and antiresorptive effect at healing sites.^21^,^22^ This mechanistic rationale may help explain why several studies included in the present review reported improved mineralization and greater socket density in simvastatin-treated sites. Nevertheless, radiographic improvement did not always translate into consistently better short-term clinical outcomes. Although several studies found no significant differences in postoperative pain, swelling, or soft-tissue healing between simvastatin-treated and control sites,^2^,^15^,^16^,^17^ some investigations reported a more favorable early postoperative course in the experimental group,^18^,^23^ whereas others described greater early discomfort or delayed initial mucosal healing despite superior radiographic bone regeneration.^19^ Therefore, the apparent osteogenic benefit of simvastatin should not be interpreted as synonymous with uniformly improved short-term clinical recovery.

The comparative evidence should also be interpreted carefully. In most studies using untreated sockets or carrier-only controls, simvastatin showed a favorable regenerative profile.^15^,^16^,^17^,^18^,^19^,^28^ However, its performance was less predictable when tested against other active interventions. Simvastatin showed better early bone regeneration than melatonin,^23^ but it did not outperform all alternative regenerative strategies. In particular, sticky bone was associated with superior postoperative and radiographic outcomes,^24^ and deproteinized bovine bone mineral with collagen remained more favorable than simvastatin-containing synthetic scaffolds in maxillary third molar ridge preservation.^12^ In addition, when 2 local simvastatin formulations were directly compared, powder and gel appeared to provide comparable regenerative effects, without significant intergroup differences.^2^ These findings suggest that simvastatin may be better interpreted as a simple osteopromotive adjunct rather than as a universally superior alternative to more established grafting materials.

Another important point concerns the marked heterogeneity of the included studies. Considerable differences were observed in study design, carrier type, comparator, follow-up duration, and outcome assessment methods. Simvastatin was delivered through gelfoam, gelatin sponge, collagen sponge, gel formulations, and scaffold-based systems, and was compared not only with spontaneous healing or passive carriers but also with melatonin, sticky bone, and synthetic or xenogeneic regenerative materials.^2^,^12^,^15^,^16^,^17^,^18^,^19^,^20^,^23^,^24^,^29^ In addition, outcome measures ranged from gray-scale histogram analysis and CBCT-derived density parameters to clinical variables such as pain, swelling, wound healing, and trismus. This cautious interpretation is also consistent with the broader alveolar ridge preservation literature, in which ridge-preservation interventions generally attenuate postextraction dimensional loss, although clinical heterogeneity remains substantial across materials, carriers, and outcome measures.^14^,^30^,^31^,^32^ Moreover, a recent broader systematic review on local simvastatin after tooth extraction also reported a favorable trend for bone regeneration, while emphasizing heterogeneity in outcome assessment and limited certainty regarding patient-centered outcomes.^33^

The methodological quality of the available evidence further limits the strength of the conclusions. As shown in Figures 2 and 3, most randomized studies presented some concerns overall and 2 were judged at high risk of bias, whereas the nonrandomized comparative study showed a serious overall risk of bias. Therefore, the favorable trend observed for simvastatin should be interpreted prudently. Moreover, most studies were based on small samples and relatively short follow-up periods, which restrict the possibility of determining whether the early radiographic advantages are maintained over time and translated into clinically meaningful long-term bone quality. Future randomized clinical trials should therefore include larger samples, longer follow-up, and more standardized imaging and clinical outcome measures to better define the true regenerative value of locally delivered simvastatin in third molar surgery.

## CONCLUSION

Within the limitations of the available evidence, locally delivered simvastatin may be associated with improved radiographic or tomographic indicators of hard-tissue healing after third molar extraction, particularly when compared with untreated sites or passive carriers alone.^15^,^16^,^17^,^18^,^19^,^23^,^28^ However, the available evidence is heterogeneous and methodologically limited, and the observed advantages mainly concern surrogate imaging-based outcomes rather than consistent patient-centered benefits. The effects on postoperative pain, swelling, and soft-tissue healing were less consistent across studies. Therefore, the current evidence does not yet allow firm conclusions regarding the real clinical advantage of simvastatin in postextraction socket management. Further well-designed randomized clinical trials with standardized outcome measures, larger samples, and longer follow-up are required before clinical recommendations can be established.

## Supplementary Material
