# Supplementary material for: Local Simvastatin for Hard-Tissue Healing After Third Molar Extraction: A Systematic Review
Source: J Craniofac Surg. 2026 Aug 25;37(11-12):3382–6. doi: 10.1097/SCS.0000000000013261 (PMC13764261; doi:10.1097/SCS.0000000000013261)
Supplement: Supplementary file 1 [file scs-37-03382-s001.docx]

Supplemental Table 1 Search Strategy

**PubMed**

Search: (simvastatin[Title/Abstract] OR "Simvastatin"[Mesh]) AND ("Molar, Third"[Mesh] OR "third molar"[Title/Abstract] OR "third molars"[Title/Abstract] OR "mandibular third molar"[Title/Abstract] OR "maxillary third molar"[Title/Abstract] OR "wisdom tooth"[Title/Abstract] OR "wisdom teeth"[Title/Abstract]) AND (extraction[Title/Abstract] OR extracted[Title/Abstract] OR "tooth extraction"[Mesh] OR "tooth extraction"[Title/Abstract] OR socket[Title/Abstract] OR "extraction socket"[Title/Abstract] OR "post-extraction"[Title/Abstract])

**Scopus**

TITLE-ABS-KEY (simvastatin) AND TITLE-ABS-KEY ("third molar" OR "third molars" OR "mandibular third molar" OR "maxillary third molar" OR "wisdom tooth" OR "wisdom teeth”) AND TITLE-ABS-KEY (extraction OR extracted OR "tooth extraction" OR socket OR "extraction socket" OR "post-extraction")

**Web of Science**

TS=(simvastatin) AND TS= ("third molar" OR "mandibular third molar" OR "maxillary third molar" OR "wisdom tooth") AND TS=(extraction)

**LILACS**

(simvastatin) AND ("third molar" OR "third molars" OR "wisdom tooth" OR "wisdom teeth") AND (extraction OR "tooth extraction" OR socket OR "extraction socket" OR "post-extraction")
