# Supplementary material for: Local Simvastatin for Hard-Tissue Healing After Third Molar Extraction: A Systematic Review
Source: J Craniofac Surg. 2026 Aug 25;37(11-12):3382–6. doi: 10.1097/SCS.0000000000013261 (PMC13764261; doi:10.1097/SCS.0000000000013261)
Supplement: Supplementary file 2 [file scs-37-03382-s002.docx]

Supplemental Table 2 Summary of included studies

| **Study** | **Country** | **Design / sample** | **Teeth / sites** | **Intervention** | **Comparator** | **Follow-up / outcomes** | **Main findings** |
| --- | --- | --- | --- | --- | --- | --- | --- |
| **Tidke et al., 2025** | India | Randomized, split-mouth, single-blind, single-center trial; **10 patients** | Bilateral impacted mandibular third molars (**20 sockets**) | **Simvastatin 10 mg powder** placed in socket | **Simvastatin 10 mg gel** (2% hydrogel/chitosan-based local formulation) | Pain: day 1 and 7; wound healing: day 7; RVG gray-scale values: immediate postop, 1, 4, 8, and 12 weeks | Both formulations were associated with progressive bone fill over time; no significant intergroup difference was reported, suggesting comparable regenerative performance between powder and gel. |
| **Degala and Bathija, 2018** | India | Randomized, split-mouth, single-blind, single-center trial; **30 patients** | Bilaterally impacted mandibular third molars (**60 sockets**) | **Simvastatin 10 mg + Gelfoam** | **Gelfoam + saline** | Pain and swelling; IOPAR gray-scale analysis at 1, 4, 8, and 12 weeks; CBCT in every fifth patient at 12 weeks | Mean gray-level histogram values were significantly higher in simvastatin sites at all time points; CBCT findings further supported enhanced bone regeneration in the test sites. |
| **Harsha et al., 2023** | India | Randomized controlled trial with split-mouth allocation; **50 patients** | Bilateral mandibular third molars (**100 sockets**) | **Simvastatin 10 mg powder + Gelfoam moistened with saline** | **Gelfoam moistened with saline** | Pain and swelling: day 1 and 7; IOPA gray-scale values: 1, 4, 8, and 12 weeks; CBCT at 12 weeks | Simvastatin sites showed significantly higher radiographic gray-scale values and higher CBCT density at 12 weeks; pain and swelling differences were not significant; no major postoperative complications were reported. |
| **Chauhan et al., 2015** | India | Prospective clinical pilot study; **30 patients** | Bilateral similarly impacted mandibular third molars (**60 sockets**) | Local **simvastatin** placement in extraction sockets | Control sockets without simvastatin | Serial IOPA assessment and gray-scale histogram analysis over **3 months**; clinical comparison with controls | The simvastatin sites showed faster osseous regeneration and more favorable gray-level histogram values than control sites. |
| **Deepanjali et al., 2023** | India | Prospective, randomized, split-mouth, single-blind study; **15 patients** | Bilateral impacted mandibular third molars (**30 sockets**) | **Simvastatin 10 mg + gelatin sponge/Gelfoam** | **Gelatin sponge/Gelfoam alone** | Pain, swelling, infection: days 1, 3, and 7; CBCT bone density at **6 months** | Mean gray value was significantly higher in simvastatin sites (**429.133**) than in control sites (**310.153**), indicating improved bone regeneration at 6 months. |
| **Mahdi and Al-Adili, 2021** | Iraq | Prospective comparative randomized clinical study; **24 patients / 32 cases** | Surgically extracted mandibular third molars | **Simvastatin 10 mg + Gel foam** | **Gel foam alone** | CBCT voxel-value bone density at **3 months**; pain, swelling, mouth opening, complications | Bone density at 3 months was significantly higher in the simvastatin group (**486.78 VV**) than in controls (**327.00 VV**); authors also reported less postoperative pain in the simvastatin group. |
| **Diniz et al., 2022** | Brazil | Single-blind, split-mouth, randomized controlled trial; **29 enrolled, 22 completed** | Impacted mandibular third molars (**44 sockets analyzed**) | **Absorbable collagen sponge + simvastatin 20 mg** | **Clot alone** | CBCT immediately postop and at **3 months** (BV, BV/TV, Tb.Th, Tb.Sp, gray scale); pain, swelling, Landry index | Simvastatin improved BV, BV/TV, and trabecular bone parameters, but was associated with greater early pain, edema, and worse early soft-tissue healing; no adverse effects or long-term mucosal differences were observed. |
| **Sinha and Das, 2025** | India | Prospective, randomized, single-blind, split-mouth study; **30 patients** | Bilateral mandibular third molar extractions (**60 sockets**) | **Simvastatin 10 mg in gelatin sponge** | **Melatonin 3 mg in gelatin sponge** | Pain: postop day 1 and week 1; Landry healing index: weeks 1, 4, 8, and 12; RVG gray-scale values: baseline, 1, 4, 8, and 12 weeks | Simvastatin showed significantly higher early gray-scale values (weeks 1 and 4) and lower week-1 pain scores than melatonin; no significant difference in soft-tissue healing was observed. |
| **Oliveira et al., 2017** | Brazil | Prospective, controlled, randomized, split-mouth clinical trial; **13 patients / 26 sockets** | Bilateral **maxillary** third molar sockets | **PLGA/HA/β-TCP scaffold with 2.0% simvastatin** (PLGA/HA/S; **8 sites**) | **PLGA/HA scaffold**, **DBBM-C**, and **spontaneous healing** | Clinical complications and CBCT evaluation in a subset at **3 months** | The simvastatin-containing scaffold showed fewer complications than the same scaffold without simvastatin, but DBBM-C performed better overall; scaffold-based approaches were associated with graft loss and more complications than expected. |
| **Hashmi et al., 2025** | India | Comparative clinical trial; **22 patients / 44 surgery sites** | Impacted mandibular third molar sockets | **Simvastatin 10 mg with Gel foam** | **Sticky bone (xenograft-based graft)** | Pain, edema, mouth opening, gingival healing: day 3, day 7, and 1 month; bone regeneration and CBCT density: 1 and 3 months | Sticky bone showed better postoperative pain, swelling, mouth opening, bone regeneration, and bone density than simvastatin; gingival healing was not significantly different between groups. |
